# p62/SQSTM1 promotes rapid ubiquitin conjugation to target proteins after endosome rupture during xenophagy

**DOI:** 10.1002/2211-5463.12385

**Published:** 2018-02-07

**Authors:** Megumi Tsuchiya, Hidesato Ogawa, Takako Koujin, Chie Mori, Hiroko Osakada, Shouhei Kobayashi, Yasushi Hiraoka, Tokuko Haraguchi

**Affiliations:** ^1^ Graduate School of Frontier Biosciences Osaka University Suita Japan; ^2^ Advanced ICT Research Institute Kobe National Institute of Information and Communications Technology Kobe Japan

**Keywords:** artificial beads, autophagy, bacterial infection, LC3, p62, ubiquitination

## Abstract

Autophagy is a bulk degradation pathway, and selective autophagy to remove foreign entities is called xenophagy. The conjugation of ubiquitin to target pathogens is an important process in xenophagy but when and where this ubiquitination occurs remains unclear. Here, we analyzed the temporal sequence and subcellular location of ubiquitination during xenophagy using time‐lapse observations, with polystyrene beads mimicking invading pathogens. Results revealed accumulation of a ubiquitination marker around the beads within 3 min after endosome rupture. Recruitment of ubiquitin to the beads was significantly delayed in p62‐knockout murine embryonic fibroblast cells, and this delay was rescued by ectopic p62 expression. Ectopic expression of a phosphorylation‐mimicking p62 mutated at serine residue 405 (equivalent to human serine residue 403) rescued this delay, but its unphosphorylated form did not. These results indicate that ubiquitination mainly occurs after endosome rupture and suggest that p62, specifically the phosphorylated form, promotes ubiquitin conjugation to target proteins in xenophagy.

**Abbreviations**
CLEM: correlative light–electron microscopy
EM: electron microscopy
FM: fluorescence microscopy
GAPDH: glyceraldehyde 3‐phosphate dehydrogenase
KO: knockout
LC3: microtubule‐associated protein light chain 3
MEF: murine embryonic fibroblast
NDP52: nuclear dot protein 52
SQSTM1: sequestosome‐1

Autophagy is a cytosolic bulk degradation pathway that facilitates the recycling of biomolecules through nonspecific degradation of proteins and organelles under nutrient starvation conditions 1, 2, 3. Additionally, autophagy plays an important defensive role against infection by pathogens, such as *Salmonella typhimurium* or *Mycobacterium tuberculosis*, in fed cells 4, 5 as part of a starvation‐independent autophagic defense system called xenophagy (digestion of foreign pathogens). Several distinct xenophagy pathways were recently identified showing that xenophagy can be triggered by host‐mediated post‐translational modifications of either bacteria‐containing vacuoles or cytosolic bacteria 4, 6. Among various post‐translational modifications, ubiquitination is one of the most common modifications in these pathways.

Ubiquitination plays a role in recruiting autophagy receptors, including p62/sequestosome‐1 (SQSTM1), optineurin (OPTN), nuclear dot protein 52 (NDP52), and a neighbor of the BRCA1 gene (NBR1), to pathogens or the damaged membranes of compartment‐containing pathogens 7, 8. These autophagy receptors initiate isolation membrane formation by bridging ubiquitinated substrates and microtubule‐associated protein light chain 3 (LC3) via their LC3‐interacting region and ubiquitin‐binding domain, respectively 9, 10, 11, 12. LC3 associates with the autophagosome and is required to elongate the isolation membrane and recruit proteins necessary for autophagy progression 13.

Ubiquitination of pathogens or host proteins can occur at any step of xenophagy 7. During bacterial infection, invading bacteria perturb the ubiquitination process in various ways depending on the bacterial species to survive and proliferate in infected cells 14, 15. Therefore, discriminating between bacteria‐induced reactions and the response of host cells during xenophagy is difficult. To overcome this difficulty, we developed an experimental system using artificial beads conjugated with pHrodo as a substitute for invading pathogens. pHrodo is a pH‐sensitive fluorogenic dye that is almost nonfluorescent under neutral pH conditions, but becomes highly fluorescent in acidic conditions 16. Therefore, pHrodo‐conjugated beads serve as an indicative marker for endosome rupture, as they emit fluorescence in the acidic endosome and lose fluorescence when exposed to the neutral environment of the cytosol following endosomal membrane rupture. Using this system, the beads are first incorporated into the cells through macropinocytosis, are entrapped in acidic endosomes, escape to the cytoplasm upon endosome rupture, and are finally entrapped by autophagosomes 17. This experimental system allows the monitoring of the host‐cell responses in the absence of bacteria‐induced reactions.

Using this system, we assessed the ubiquitination process associated with invading entities during xenophagy by observing the dynamic behavior of GFP‐fused ubiquitin in living cells using fluorescence microscopy (FM) and correlative light–electron microscopy (CLEM). Furthermore, we observed ubiquitin dynamics in p62‐depleted cells using the same system. These results show that ubiquitination mainly occurs in ruptured endosomal membranes after endosome rupture and that the ubiquitination process is promoted by the autophagy receptor protein p62.

## Materials and methods

### Plasmids

To construct the PBEF1‐EGFP‐MCS‐neo vector, the DNA fragment encoding GFP was amplified from the pEGFP‐C1 vector (PT3028‐5; Clontech Laboratories, Mountain View, CA, USA) using PCR and the primers 5′‐TGTGACCGGGCGCCTACTATGGTGAGCAAGGGCGAGGAGCT‐3′ and 5′‐CGAATTCGCTAGCTCTAGACTTGTACAGCTCGTCCATGC‐3′ and inserted into the PBEF1‐MCS‐IRES‐neo cDNA expression vector (PB533A‐2; System Biosciences, Palo Alto, CA, USA) after digestion with *Xba*I. To construct the PBEF1‐EGFP‐Ubwt‐neo vector (GFP‐Ubwt‐neo), the DNA fragment encoding human wild‐type (WT) ubiquitin (Ubwt) was amplified from the cDNA of HeLa cells using PCR and the primers 5′‐TCTAGAGCTAGCGAATTCATGCAGATCTTCGTGAAGACTCTGA‐3′ and 5′‐TCCGATTTAAATTCGAATTCTTACCCACCTCTGAGACGGAGTAC‐3′ and inserted into the PBEF1‐EGFP‐MCS‐neo vector after digestion with *Eco*RI. To construct the PBEF1‐EGFP‐Ubunconj‐neo vector (GFP‐Ubunconj‐neo), the DNA fragment encoding a ubiquitin mutant (Ubunconj) was amplified from pGEX6P‐1‐hUbiquitin K0 (kindly provided by Yasushi Saeki, Tokyo Metropolitan Institute of Medical Science, Tokyo, Japan) using PCR and the primers 5′‐CTAGAGCTAGCGAATTCATGCAGATTTTCGTGAGAACCC‐3′ and 5′‐TCCGATTTAAATTCGAATTCTTAAACACCACGAAGTCTCA‐3′ and inserted into the PBEF1‐EGFP‐MCS‐neo vector after digestion with *Eco*RI. Ubunconj was mutated at all lysine residues, which were substituted with arginine residues (K0‐Ub) 18, and at the C‐terminal glycine residue, which was substituted with a valine residue (G76V) 19, 20, thereby rendering a conjugation‐deficient monomeric ubiquitin. The PBEF1‐EGFP‐Ubwt‐puro vector (GFP‐Ubwt‐puro) was prepared by replacing the coding region of a neomycin‐resistance gene in the PBEF1‐EGFP‐Ubwt‐neo vector with that of a puromycin‐resistance gene, because the original p62‐knockout (KO) murine embryonic fibroblast (MEF) cells contained the neomycin‐resistance gene. The puromycin‐resistance gene sequence was amplified from the pBabe‐puro plasmid 21 by PCR using the following primers: 5′‐TCTAGAGCTAGCGAATTCATGACCGAGTACAAGCCCACGGT‐3′ and 5′‐ACAACCATGGCGTCCGGAATGACCGAGTACAAGCCCA‐3′. To construct a DNA plasmid encoding mCherry‐Ubwt, the PBEF1‐mCherry‐Ubwt‐puro vector (mCherry‐Ubwt‐puro) was prepared by replacing the coding region for EGFP cDNA in the PBEF1‐EGFP‐Ubwt‐puro vector with that of mCherry cDNA. The mCherry sequence was amplified by PCR using the following primers: 5′‐ TGTGACCGGGCGCCTACTATGGTGAGCAAGGGCGAGGAGG‐3′ and 5′‐CGAATTCGCTAGCTCTAGACTTGTACAGCTCGTCCATGCC‐3′. The expression plasmid for murine p62 (PEF1‐mp62‐zeo) was prepared as previously described 22. To construct the expression plasmids for the p62S405A mutant involving substitution of the serine at residue 405 with alanine, PCR was performed using primers 5′‐CTCTCCCAGATGCTGGCCATGGGTTTCTCGGATGAA‐3′ and 5′‐TTCATCCGAGAAACCCATGGCCAGCATCTGGGAGAG‐3′. For the p62S405E mutant involving substitution of the same serine with glutamic acid, PCR was performed using the primers 5′‐CTCTCCCAGATGCTGGAGATGGGTTTCTCGGATGAA‐3′ and 5′‐TTCATCCGAGAAACCCATCTCCAGCATCTGGGAGAG‐3′. The mutant cDNA were amplified by PCR using the primers 5′‐TCTAGAGCTAGCGAATTCATGACCGAGTACAAGCCCACGGT‐3′ and 5′‐ACAACCATGGCGTCCGGAATGACCGAGTACAAGCCCA‐3′ and inserted into PEF1‐mp62‐zeo vectors.

### Cell strains

Murine embryonic fibroblast cells and p62‐KO MEF cells (p62^−/−^ cells) were kindly provided by Tetsuro Ishii 22, 23. MEF cells stably expressing GFP‐LC3 were kindly provided by Tamotsu Yoshimori (Osaka University, Osaka, Japan). To obtain MEF cells stably expressing GFP‐Ubwt or GFP‐Ubunconj, MEF cells were transfected with plasmids encoding GFP‐Ubwt‐neo (GFP‐Ubwt MEF cells) or GFP‐Ubunconj‐neo (GFP‐Ubunconj MEF cells) and cultured in the presence of G418 disulfate (16513‐26; Nacalai Tesque, Kyoto, Japan). To obtain MEF cells stably expressing GFP‐LC3 and mCherry‐Ubwt, GFP‐LC3 MEF cells were transfected with the DNA plasmid encoding mCherry‐Ubwt‐puro (GFP‐LC3/mCherry‐Ubwt MEF cells) and cultured in the presence of puromycin (ant‐pr‐1; InvivoGen, San Diego, CA, USA). To obtain p62‐KO MEF cells stably expressing GFP‐Ubwt (p62‐KO/GFP‐Ubwt MEF cells), p62‐KO MEF cells were transfected with the plasmid encoding GFP‐Ubwt‐puro and cultured in the presence of puromycin. To obtain the p62‐KO MEF cells expressing either p62 WT, p62S405A mutant, or p62S405E mutant, p62‐KO MEF cells were transfected with plasmids encoding either murine p62, p62S405A, or p62S405E and cultured in the presence of Zeocin. Clones stably expressing the respective proteins were examined for protein expression by western blot.

### Cell culture

Cells were maintained in Dulbecco's modified Eagle medium (DMEM; D6429; Sigma‐Aldrich, St. Louis, MO, USA) supplemented with 10% fetal bovine serum and 1× penicillin–streptomycin–glutamine (161‐23201; Wako Pure Chemical Industries, Osaka, Japan) in the presence of 5% CO_2_ at 37 °C. One day before incorporating the beads, cells were seeded onto 35‐mm glass‐bottom culture dishes (P35G‐1.5‐10‐C; MatTek, Ashland, MA, USA) at a density of 1.5 × 10^5^ cells per dish in the absence of antibiotics.

### pHrodo‐conjugated beads

pHrodo‐conjugated beads were prepared, as previously described 17. Briefly, Dynabeads M‐270 Streptavidin (DB65306; Invitrogen, Carlsbad, CA, USA) were washed three times with PBS and resuspended in 100 mm sodium bicarbonate buffer (pH 8.5) to an appropriate concentration (typically a 1 : 10 or 1 : 20 dilution). pHrodo succinimidyl ester (P36600; Invitrogen) was then added to the bead suspension and incubated in sodium bicarbonate buffer for 1 h at room temperature (about 25 °C). After the conjugation reaction, the beads were washed with sodium bicarbonate buffer and suspended in PBS.

### Incorporation of beads into living cells

Beads were incorporated into cells as previously described 24. Briefly, 1 day before bead incorporation, cells were seeded onto 35‐mm glass‐bottom culture dishes (P35G‐1.5‐10‐C; MatTek) at a density of 1.5 × 10^5^ cells per dish in the absence of antibiotics. Transfection reagent‐coated beads were prepared by mixing pHrodo‐conjugated beads with Effectene transfection reagent (301425; Qiagen K.K., Tokyo, Japan) according to the manufacturer's instructions, except that the bead suspension was used instead of DNA solution. The resulting bead mixture (~ 10 μL) was mixed with 90 μL of the growth medium and added to the cells by replacing the medium. After incubation for 1 h at 37 °C in a CO_2_ incubator, cells were washed twice with the fresh growth medium to remove unattached beads and further incubated for the time indicated in each experiment.

### Time‐lapse imaging

Cells were treated with 100 ng·mL^−1^ Hoechst33342 (B2261; Sigma‐Aldrich) for 15 min to stain chromosomes as previously described 25. After replacing the culture medium with fresh medium not containing phenol red, time‐lapse observation was performed using an oil‐immersion objective lens (UApo40/NA1.35; Olympus, Tokyo, Japan) on a DeltaVision microscope system (GE Healthcare Life Sciences Japan, Tokyo, Japan) placed in a temperature‐controlled room (37 °C) as previously described 25.

The fluorescence intensity around the beads was quantified using the fiji software suite (imagej; National Institutes of Health, Bethesda, MD, USA). The fluorescence intensity of the region (18 pixels square) surrounding the beads was measured, and the background fluorescence intensity of a region with no beads (18 pixels square) in the same cell was subtracted. The fluorescence intensity was plotted as a function of time.

### CLEM

Correlative light–electron microscopy was performed as previously described 26. Briefly, cells were fixed with 2.5% (w/v) glutaraldehyde for 1 h. Z‐stack optical images (typically 40–60 focal planes at 0.2‐μm intervals) were obtained using the Olympus oil‐immersion objective lens (PLAPON60xOSC/NA1.40) on the DeltaVision microscope system and subjected to deconvolution using standard software installed on the microscope system. After fluorescence imaging, samples were postfixed with 1% OsO_4_ (3002; Nisshin EM, Tokyo, Japan), stained with 2% (w/v) uranyl acetate (8473–1M; Wako Pure Chemical Industries) for 1 h, dehydrated, and embedded in Epon812 (T024; TAAB Laboratory Equipment, Ltd., Reading, UK). Ultrathin sections (80 nm thick) were prepared using an ultramicrotome (Leica Microsystems, Wetzlar, Germany) and stained with 4% uranyl acetate, followed by a commercial ready‐to‐use solution of lead citrate (18‐0875‐2; Sigma‐Aldrich). Electron microscopy (EM) images were acquired using a JEM‐1400 electron microscope (80 kV; JEOL, Tokyo, Japan), and EM images were overlaid with the corresponding fluorescence images.

### Western blot

To prepare whole‐cell extracts, cells (5 × 10^5^) were harvested and resuspended in 250 μL of 1× NuPAGE LDS sample buffer (NP0008; Thermo Fisher Scientific K.K., Yokohama, Japan). Whole‐cell extracts were sonicated briefly to reduce viscosity, and 10 μL of the extract was subjected to electrophoresis on 4–12% Bis–Tris NuPAGE gels (NP0321; Thermo Fisher Scientific K.K.). Proteins were transferred to polyvinylidene fluoride membranes and probed using rabbit anti‐GFP (600‐401‐215; Rockland Immunochemicals, Pottstown, PA, USA), anti‐p62/SQSTMI (P0067; Sigma‐Aldrich), or anti‐glyceraldehyde 3‐phosphate dehydrogenase (anti‐GAPDH) antibody (14C10; Cell Signaling Technology, Danvers, MA, USA) and secondary antibodies conjugated to horseradish peroxidase (NA9340; GE Healthcare Life Sciences). Protein bands were stained with ImmunoStar Zeta (295‐72404; Wako Pure Chemical Industries) and detected by chemiluminescence using a ChemiDoc MP imaging system (Bio‐Rad, Tokyo, Japan).

### Indirect immunofluorescence microscopy

Murine embryonic fibroblast cells grown in a glass‐bottom dish were fixed with 4% PBS for 15 min at room temperature. After washing with 0.1% Tween 20 in PBS (PBST), cells were permeabilized with 0.5% Triton X‐100 in PBS for 5 min at room temperature and washed with PBST three times. The cells were then blocked with Blocking One solution (03953‐95; Nacalai Tesque) for 30 min at 4 °C, washed once with PBST, and incubated with a rabbit polyclonal anti‐p62 antibody (P0067; Sigma‐Aldrich) in Can Get Signal Immunostain Solution A (NKB‐501; Toyobo, Osaka, Japan) for 2 h, followed by extensive washes and incubation with Cy3‐conjugated donkey anti‐rabbit IgG antibody (AP182C; Life Technologies, Carlsbad, CA, USA) for 1 h. After washing with PBST three times, cells were stained with 4′,6‐diamidino‐2‐phenylindole (DAPI) and subjected to FM. For observation, an oil‐immersion objective lens (PLAPON60xOSC/NA1.40; Olympus) on the DeltaVision microscope system (GE Healthcare Life Sciences) was used as previously described 25.

### Statistical analysis

The *P*‐values were obtained by Kruskal–Wallis test using graphpad Prism 7 software (GraphPad Software, Inc, La Jolla, CA, USA).

## Results and Discussion

### Ubiquitin assembles around the invading beads following endosome rupture

**Figure 1:**
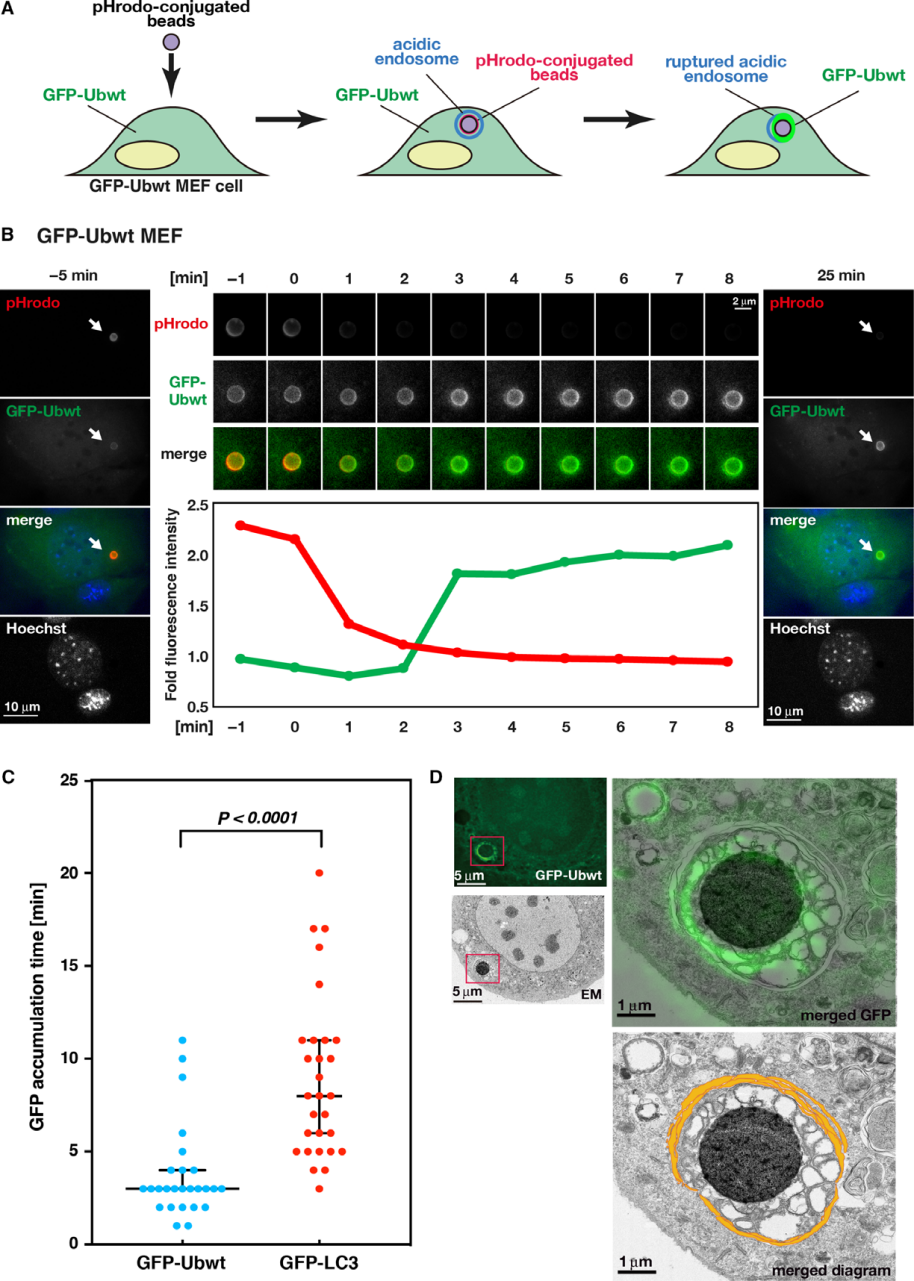
Ubiquitination occurs around the beads after endosome rupture. (A) Schematic diagram of the experimental system using pHrodo‐conjugated beads. Beads transferred to GFP‐Ubwt MEF cells (left) become pHrodo positive (red) upon being engulfed by acidic endosomes (middle). After endosome rupture, the beads lose the red fluorescence upon exposure to the cytosol at neutral pH. Then, the beads become GFP positive (green) in the presence of GFP‐Ubwt (right). (B) Time‐lapse images of pHrodo and GFP‐Ubwt signals in GFP‐Ubwt MEF cells. Images were obtained every minute for 30 min. Selected time frames are displayed. The 0‐min time point represents the time when the pHrodo signal began to decrease upon endosome rupture. The left and right panels show overview images of the cell at −5 and 25 min, respectively, during time‐lapse observation. Blue, green, and red colors in the merged images represent Hoechst33342, GFP‐Ubwt, and pHrodo signals, respectively. Arrows indicate the position of the beads. Scale bar, 10 μm. The middle upper panels show time‐lapse images of the pHrodo and GFP‐Ubwt signals around a single pHrodo bead in the same cell. The lower graph shows time‐dependent changes in the relative fluorescence intensity of pHrodo (red) and GFP‐Ubwt (green) around the beads indicated in the upper panels. Scale bar, 2 μm. (C) Statistical analysis was performed for the timing of GFP‐signal accumulation around the beads after the loss of pHrodo signals in cells expressing GFP‐Ubwt (left) and GFP‐LC3 (right; also see Fig. S1A). The median times were 3 min for GFP‐Ubwt and 8 min for GFP‐LC3. Statistical significance (*P* < 0.0001) of the differences in timing between GFP‐Ubwt and GFP‐LC3 using 26 and 29 beads, respectively. Error bars indicate 95% confidence intervals. (D) CLEM analysis of beads in GFP‐Ubwt MEF cells. The left panels show the fluorescence image of GFP‐Ubwt (upper) and the electron micrograph (lower) of the same GFP‐Ubwt MEF cell. Scale bars, 5 μm. The right panels show high‐magnification EM images of the boxed region in the overview image (left panels). The FM image of the same cell is superimposed in green (upper); the positions of isolation membranes are marked by orange lines (lower). Scale bars, 1 μm.

To investigate when and where ubiquitination occurs during xenophagy, we developed the following experimental system using polystyrene beads. The beads were incorporated into MEF cells that expressed a GFP‐fused WT ubiquitin (GFP‐Ubwt MEF cells) as a substrate for xenophagy, and the assembly of GFP‐Ubwt at the position of the beads was monitored using FM. The beads were preconjugated with pHrodo dye, which emits fluorescence only under acidic pH conditions, and loaded into GFP‐Ubwt MEF cells (Fig. 1A). The pHrodo‐conjugated beads emit fluorescence in the acidic endosome and lose their fluorescence when exposed to the cytosol, thereby serving as a marker of endosomal membrane rupture 17.

Using this experimental system, we performed time‐lapse observations of the pHrodo‐conjugated beads. The results showed that the beads initially became pHrodo fluorescence positive, suggesting bead incorporation into the acidic endosome, as previously observed (Fig. 1B, left panel) 17. The beads then lost the fluorescence (compare the 0‐min time point with that for 1 min; Fig. 1B, middle), indicating that the endosomal membrane had ruptured between 0 and 1 min. Following loss of the pHrodo signal, the fluorescence intensity of GFP‐Ubwt increased (Fig. 1B, middle) and was maintained over 30 min. The timing of ubiquitin recruitment to the beads was ~ 3 min (median) after pHrodo fluorescence began to decrease [mean and standard error of the mean (SEM): 3.8 ± 0.5 min, *n* = 26 beads; Fig. 1C]. Because ubiquitin conjugation occurs prior to LC3 assembly in HeLa cells 27, we evaluated the timing of LC3 assembly to the beads in MEF cells expressing GFP‐LC3 (Fig. S1A) and compared the timing with that of GFP‐Ubwt in MEF cells (Fig. 1B). GFP‐LC3 assembled around the beads at ~ 8 min (median) after pHrodo fluorescence began to decrease (mean and SEM: 8.9 ± 0.8 min, *n* = 29 beads), indicating that ubiquitin assembly to the beads occurred before LC3 assembly in MEF cells, similar to that observed in HeLa cells 27. To confirm this result, we also examined the timings of assembly of ubiquitin and LC3 to the beads in cells expressing both mCherry‐Ubwt and GFP‐LC3 and found that mCherry‐Ubwt assembled to the beads several minutes earlier than GFP‐LC3 (Fig. S1B), indicating that ubiquitin assembly to the substrates occurred prior to LC3 assembly.

To elucidate the subcellular structures targeted by ubiquitin during xenophagy, we observed GFP‐Ubwt‐positive beads using CLEM 26: CLEM is a method by which the same specimen is sequentially observed using FM and EM, and then, the correlation between the FM and EM images is determined (Fig. 1D). EM images showed intricate vesicular structures surrounding the beads (diameter: 200–500 nm). Additionally, these structures were surrounded by multilayered, typical isolation membranes (Fig. 1D, bottom), indicating that the beads had been engulfed by autophagosomes. The GFP signals overlapped with signals from the surrounding vesicular membranes located inside the autophagosomes (Fig. 1D, right upper panel). Because proteins on the endosomal membrane become ubiquitination targets during bead‐induced xenophagy 27, these vesicular membranes were likely membranes originating from endosomes. Therefore, our results suggested that the endosomal membranes were ubiquitinated after endosome rupture. Collectively, these data suggest the following sequence of events occurs during bead‐induced xenophagy: (a) The invading beads enter the endosomes and escape after endosome rupture, (b) ubiquitin associates with target molecules in remnants of the endosomal membrane around the beads, and (c) autophagosomes engulf the ubiquitinated membranes surrounding the invading beads. These events likely mimic cellular responses to invading pathogens.

### Conjugation‐deficient ubiquitin does not accumulate around the beads

**Figure 2:**
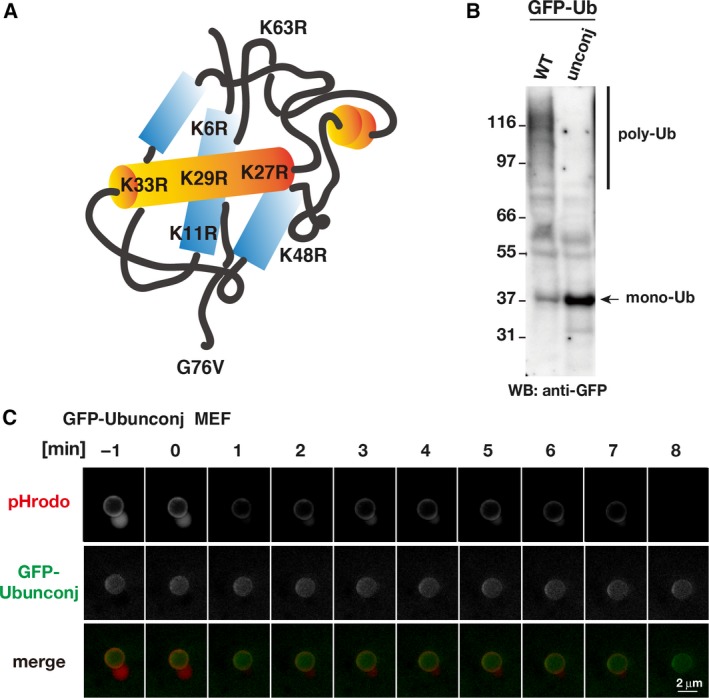
The conjugation‐deficient ubiquitin mutant does not accumulate around beads. (A) Schematic diagram of the Ubunconj construct involving mutation of all seven lysine residues (K) to arginine (R). (B) Ubiquitin polymeric/monomeric status analyzed by western blot of whole‐cell extracts from GFP‐Ubwt (WT)‐ and GFP‐Ubunconj (unconj) MEF cell lines. (C) Time‐lapse images of pHrodo and GFP‐Ubunconj fluorescence around a single pHrodo bead in GFP‐Ubunconj MEF cells. Images were obtained every minute for 30 min. Selected time frames are shown. The 0‐min time point represents the time when the pHrodo signal began to decrease upon endosome rupture. Red and green colors in the merged images represent pHrodo and GFP‐Ubunconj signals, respectively. Scale bar, 2 μm. A roundish bleb‐like structure next to the beads is often observed in the MEF cell lines used in this study when the beads are in the acidic endosome.

Correlative light–electron microscopy results indicated that fluorescence signals of GFP‐Ubwt clearly accumulated around the beads after endosome rupture. To examine whether ubiquitin assembly at the beads is the result of ubiquitin conjugation during xenophagy, we examined the behavior of a conjugation‐deficient ubiquitin mutant (Ubunconj; K0‐Ub G76V) in this experimental system: This mutant lacks all seven internal lysine residues and a C‐terminal glycine residue and therefore is incapable of conjugation to the target proteins (Fig. 2A) 18, 19, 20. We generated GFP‐Ubunconj MEF cells and confirmed the presence of monomeric (unconjugatable) ubiquitin (Fig. 2B, arrow) and the absence of the polymeric form by western blot (Fig. 2B). By contrast, we verified that control cells expressing GFP‐Ubwt contained a large fraction of polymeric ubiquitin (Fig. 2B). To evaluate the effect of ubiquitination on bead‐induced xenophagy, we transferred pHrodo‐conjugated beads into GFP‐Ubunconj MEF cells and monitored xenophagy of the beads using FM over 30 min. Time‐lapse observations showed early pHrodo‐positive signals (Fig. 2C), which were lost between the 0‐ and 1‐min time points (Fig. 2C) and remained absent until the end of observation (over 30 min after endosome rupture), suggesting that endosome rupture occurred at between 0 and 1 min, as expected. However, fluorescence signals of GFP‐Ubunconj remained constant at almost the background levels after the loss of pHrodo signals, indicating that conjugation‐deficient ubiquitin failed to assemble around the beads after endosome rupture. These results indicate that the assembly of WT ubiquitin around the beads after endosome rupture is mediated by ubiquitin conjugation to target proteins.

### p62 promotes ubiquitination after endosome rupture

**Figure 3:**
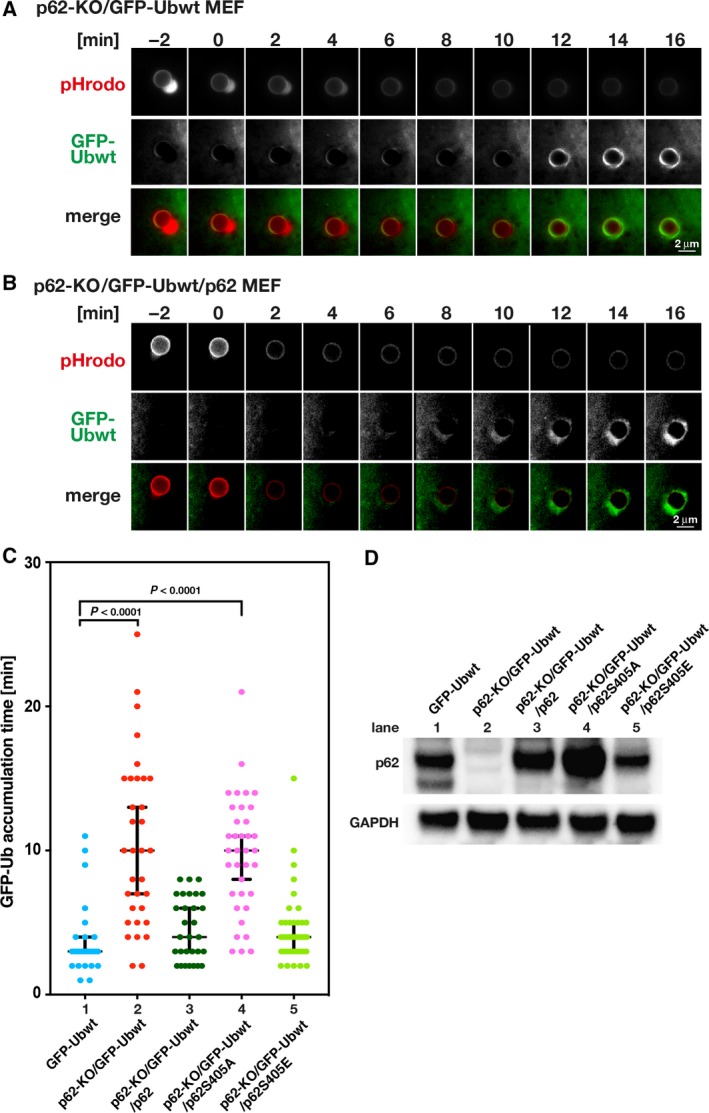
p62 affects the timing of ubiquitination. (A,B) Time‐lapse images of pHrodo and GFP‐Ubwt fluorescence around a single pHrodo bead in MEF cells. Images were obtained every minute for ~ 30 min. The panels show representative images of pHrodo and GFP‐Ubwt fluorescence in p62‐KO/GFP‐Ubwt MEF cells (A) and p62‐KO/GFP‐Ubwt/p62 MEF cells ectopically expressing p62 (B). Scale bar, 2 μm. A roundish bleb‐like structure next to the beads is often observed in the MEF cell lines used in this study when the beads are in the acidic endosome. (C) Statistical analysis was performed for the timing of GFP‐signal accumulation around the beads after the loss of pHrodo signals in the GFP‐Ubwt MEF cells that endogenously express p62 (lane 1; this was copied from Fig. 1C for ease of comparison), p62‐KO/GFP‐Ubwt cells not expressing p62 (lane 2), p62‐KO/GFP‐Ubwt/p62 cells that ectopically expressing p62 (lane 3), p62‐KO/GFP‐Ubwt/p62S405A cells ectopically expressing the p62S405A mutant (lane 4), and p62‐KO/GFP‐Ubwt/p62S405E cells ectopically expressing the p62S405E mutant (lane 5). The median time values were 3 min for GFP‐Ubwt (*n* = 26 beads), 10 min for p62‐KO/GFP‐Ubwt (*n* = 33 beads), 4 min for p62‐KO/GFP‐Ubwt/p62 (*n* = 30 beads), 10 min for p62‐KO/GFP‐Ubwt/p62S405A (*n* = 36 beads), and 4 min for p62‐KO/GFP‐Ubwt/p62S405E (*n* = 39 beads). Statistical differences (*P* < 0.0001) were determined by the Kruskal–Wallis test. Error bars indicate 95% confidence intervals. (D) Western blot analysis of p62 in the cells indicated in (C). GAPDH was detected as a loading control. The double bands in the left lane represent products of splicing variants, as previously reported 23.

Ubiquitin and p62 concomitantly localize around transfected beads 27, and autophagy receptors, such as p62, are required as a bridge between ubiquitinated substrates and autophagy machinery 28, 29; however, the roles of these receptors in regulating the ubiquitination process during xenophagy remain unknown. To determine whether p62 depletion affects substrate ubiquitination after endosome rupture, we generated p62‐KO/GFP‐Ubwt MEF cells and observed the dynamic accumulation of GFP‐Ubwt around the beads in cells using time‐lapse FM. In an example shown in Fig. 3A, the timing of the GFP‐Ubwt accumulation in p62‐KO MEF cells was 12 min after the loss of the pHrodo signal (endosome rupture). The average time of GFP‐signal accumulation was 10 min in median (mean and SEM: 10.5 ± 1.0 min, *n* = 33 beads) in p62‐KO/GFP‐Ubwt MEF cells (lane 2 in Fig. 3C), which was slower than that in control GFP‐Ubwt MEF cells (3 min in median; mean and SEM 3.8 ± 0.5 min, *n* = 26 beads; lane 1 in Fig. 3C; also see Fig. 1C left column). Additionally, the GFP‐Ubwt signals were slightly weaker than those in control GFP‐Ubwt MEF cells (compare Figs 1B and 3A). These results indicate that the timing of GFP‐Ubwt accumulation around the beads was significantly delayed in the absence of p62 in MEF cells (Fig. 3C; compare Figs 1B and 3A) and suggests a role for p62 in recruiting ubiquitin to target sites.

To confirm this finding, we examined whether p62 ectopic expression in p62‐KO MEF cells would rescue ubiquitin recruitment. We generated p62‐KO/GFP‐Ubwt/p62 MEF cells and compared p62 expression levels in p62‐KO/GFP‐Ubwt/p62 MEF cells (ectopically expressing p62) with that in parental p62‐KO/GFP‐Ubwt MEF cells (not expressing p62) and GFP‐Ubwt MEF cells (expressing endogenous p62). Western blot analysis showed that p62 expression levels in p62‐KO/GFP‐Ubwt/p62 MEF cells were similar to those in GFP‐Ubwt MEF cells (compare lane 1 with lane 3 in Fig. 3D), although the short form of the possible splicing variant expressed in the GFP‐Ubwt MEF cells was not observed in p62‐KO/GFP‐Ubwt/p62 MEF cells (lane 3 in Fig. 3D). Time‐lapse analysis showed that the timing of GFP‐Ubwt accumulation around the beads in p62‐KO/GFP‐Ubwt/p62 MEF cells was earlier than that in p62‐KO/GFP‐Ubwt MEF cells (compare lane 3 with lane 2 in Fig. 3C), with the average timing of GFP‐signal accumulation in p62‐KO/GFP‐Ubwt/p62 MEF cells at 4 min in median (mean and SEM: 4.6 ± 0.4 min, *n* = 30 beads; lane 3 in Fig. 3C), indicating that timing was restored by p62 ectopic expression. These results strongly suggest that p62 promotes substrate ubiquitination after endosome rupture. Because phosphorylation of human p62 at serine residue 403 in the ubiquitin association (UBA) domain promotes targeting of ubiquitin proteins to the sequestosome 30, 31, we further examined whether p62 phosphorylation would be required for promoting ubiquitin conjugation at the beads during bead‐induced xenophagy. We generated two MEF cell lines expressing either murine p62S405A or p62S405E mutants (the serine residue 405 in the murine p62 corresponds to the phosphorylation site at the serine residue 403 in the human p62). p62S405A and p62S405E represent an unphosphorylated p62 mutant (incapable of ubiquitin binding) and a phospho‐mimic mutant (capable of ubiquitin binding), respectively. Expression levels and subcellular localization of these mutants were similar or comparable to those of the WT p62 (Figs 3D and S1C). Time‐lapse analysis of the timing of ubiquitin assembly to the beads after endosome rupture showed that the average timing of the GFP‐signal accumulation in p62‐KO/GFP‐Ubwt/p62S405A MEF cells was greatly reduced to 10 min in median (mean and SEM: 9.7 ± 0.7 min, *n* = 36 beads) which is similar to that in p62‐KO/GFP‐Ubwt MEF cells (compare lane 4 with lane 2 in Fig. 3C). By contrast, the timing in p62‐KO/GFP‐Ubwt/p62S405E MEF cells was 4 min in median (mean: 4.5 ± 0.4 min, *n* = 39 beads) which is similar to that in p62‐KO/GFP‐Ubwt/p62 MEF cells (compare lane 5 with lane 3 in Fig. 3C). These results indicate that phosphorylation of p62 at serine residue 405 is required for promoting ubiquitination at the ruptured endosomes during xenophagy (Fig. 3C), suggesting that phosphorylated p62 at the UBA domain promotes ubiquitination during xenophagy.

In this study, we visualized the timing of ubiquitination during xenophagy using artificial beads to mimic invading pathogens and found that ubiquitination mainly occurred after endosome rupture and that p62 promoted ubiquitination of target proteins on the ruptured endosomal membrane. Several lines of evidence regarding the role of p62 in ubiquitination supported our finding. First, p62 depletion abrogates TRAF6 (a ubiquitin E3 ligase)‐dependent polyubiquitination of nerve growth factor receptors 32, 33, 34. Second, bacteria that invade cells are coated with polyubiquitin, and p62 and NDP52 bind to the polyubiquitinated coat, resulting in recruitment of autophagic machinery 9, 10, 11, 12. Third, LRSAM1 (a ubiquitin E3 ligase) binds p62 and NDP52 and can transfer ubiquitin to bacteria *in vitro* 35. These findings along with the present study suggest that autophagy receptors, such as p62, promote ubiquitination on their target molecules through binding to ubiquitin E3 ligase, leading to rapid assembly of autophagy machinery to target sites during xenophagy. Interestingly, our observation indicates that the S405‐phosphorylated murine p62, but not unphosphorylated p62, recruits ubiquitin and promotes ubiquitin conjugation to target sites. This is consistent with a previous finding that S403‐phosphorylated human p62 enhances autophagic clearance 30, 31. Thus, our results suggest that p62 promotes rapid ubiquitin conjugation to target proteins and recruits LC3 to target sites.

## Author contributions

MT, HOg, TK, CM, and HOs performed the experiments. MT, HOg, SK, YH, and TH designed the experiments. All authors analyzed and discussed the data, and MT, HOg, YH, and TH wrote the manuscript.

## Supporting information


**Fig S1**. Assembly of GFP‐LC3 around beads incorporated into MEF cells.
