# Supplementary figures and images for: p62/SQSTM1 promotes rapid ubiquitin conjugation to target proteins after endosome rupture during xenophagy
Source: FEBS Open Bio. 2018 Feb 7;8(3):470–80. doi: 10.1002/2211-5463.12385 (PMC5832981; doi:10.1002/2211-5463.12385)

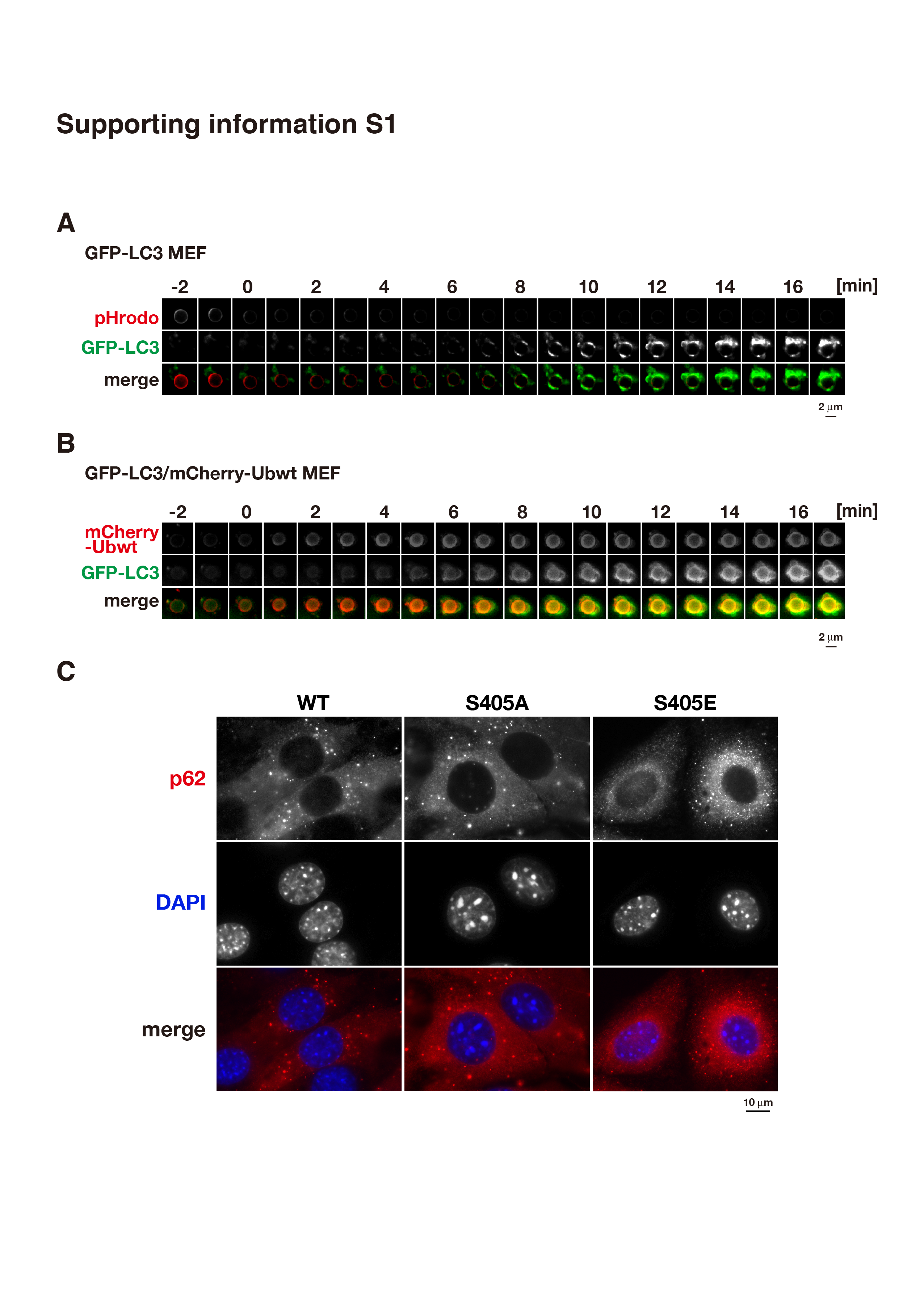

Supplement: Supplementary file 1 — Fig S1. Assembly of GFP‐LC3 around beads incorporated into MEF cells. [file FEB4-8-470-s001.tif]
