# Supplementary material for: p62/SQSTM1 promotes rapid ubiquitin conjugation to target proteins after endosome rupture during xenophagy
Source: FEBS Open Bio. 2018 Feb 7;8(3):470–80. doi: 10.1002/2211-5463.12385 (PMC5832981; doi:10.1002/2211-5463.12385)
Supplement: Supplementary file 2 [file FEB4-8-470-s002.docx]

***Fig S1. Assembly of GFP-LC3 around beads incorporated into MEF cells.*** (A) Time-lapse images of pHrodo and GFP-LC3 signals in a single GFP-LC3 MEF cell. Images were obtained every minute for ~30 min. Selected time frames are displayed. The 0-min time point represents the time when the pHrodo signal began to decrease upon endosome rupture. Red and green colors in the merged images represent pHrodo and GFP-LC3, respectively. Scale bar, 2 μm. (B) Time-lapse images of mCherry-Ubwt and GFP-LC3 signals in a single MEF cell. The 0-min time point represents the initiation of increases in mCherry signal. Red and green colors in the merged images represent mCherry-Ubwt and GFP-LC3, respectively. Scale bar, 2 μm. (C) Subcellular localization of p62, p62S405A, and p62S405E in p62-KO/GFP-Ubwt MEF cells expressing either p62 (WT), p62S405A (S405A), or p62S405E (S405E). DNA was counterstained with DAPI. Blue and red in the merged images represent signals for p62 (WT or mutants) and DNA, respectively. Scale bar, 10 μm.
